# Regulatory T cell therapy for myeloperoxidase-specific anti-neutrophil cytoplasmic antibody associated vasculitis

**DOI:** 10.3389/fimmu.2025.1675251

**Published:** 2025-11-03

**Authors:** Elean S. V. Tay, Yi T. Ting, Poh-Yi Gan, Joshua D. Ooi

**Affiliations:** Center for Inflammatory Diseases, School of Clinical Sciences at Monash Health, Monash University, Melbourne, VIC, Australia

**Keywords:** MPO-AAV, HLA, preclinical model, TCR-T and CAR-T therapy, ANCA

## Abstract

Anti-neutrophil cytoplasmic antibodies (ANCA)-associated vasculitis (AAV) is a rare autoimmune disease characterized by the inflammation of small vessels. It is most commonly caused by ANCA targeting proteinase 3 (PR3) and myeloperoxidase (MPO) which are found in neutrophil lysosomes. The most common affected organs are respiratory tracts and kidneys, though other organs can be involved too. Although the cause of disease between PR3-AAV and MPO-AAV is similar, they vary in pathogenesis. Epigenetic and genetic factors may play a role in the disease development as certain population such as Chinese with HLA-DRB1*04:05 are more prevalent in MPO-AAV patient population. The prognosis for them is usually poor, often resulting in end-stage renal failure even with existing treatment. Current treatment for AAV relies heavily on corticosteroids which are toxic for long-term usage. Hence, there is a strong need to develop new, less toxic and targeted therapy for this disease. Regulatory T cell (Treg) therapy is a new type of therapy with the potential to specifically re-establish tolerance to the target autoantigen (MPO or PR3). This review will delve into the pathogeneses of AAV and discuss the potential of using genetically engineered Tregs to treat the cause of disease.

## Anti-neutrophil cytoplasmic antibodies-associated vasculitis

AAV is a rare autoimmune disease that causes inflammation and subsequently damage to the small vessels (1–3). The main characteristic of the disease is the pauci-immune state of deposition of immunoglobulin within the small vessels of the glomeruli, which stands out as a subgroup of the small vessel vasculitides (1–3). The common affected organs include the respiratory tract, lungs and kidney. The underlying causes are autoantibodies to the neutrophil proteins; particularly proteinase 3 (PR3) and myeloperoxidase (MPO), however, other neutrophil proteins including lysosome-associated membrane protein 2 (LAMP-2) and elastase are involved to a lesser extent.

## Classification and clinical presentation

AAV can be subclassified into granulomatosis with polyangiitis (GPA) (previously known as Wegener’s granulomatosis), microscopic polyangiitis (MPA) and eosinophilic GPA (EGPA) (previously known as Churg-Strauss syndrome) according to the 2012 Revised Chapel Hill Consensus Conference (4). GPA is presented with necrotizing vasculitis with extravascular granulomatosis that usually involves respiratory tract and kidney whereas MPA is like GPA, but in the absence of granulomatosis. EGPA is characterized with necrotizing granulomatous vasculitis in the presence of eosinophilia, and it usually involves the respiratory tract with an association with asthma. A famous disease manifestation is the detection of little or no deposition of immune complexes in the affected tissue area. This results in an interesting disease presentation of pauci-immune necrotizing crescentic glomerulonephritis (NCGN). The absence of the immune complexes is due to the release of elastase from the dead neutrophils, digesting the immunoglobulin (5).

Furthermore, PR3-ANCA is usually associated with GPA (around 85% of patients) whereas MPO-ANCA is more frequently associated with MPA (around 60% of patients) (6). The titres of ANCA do not correlate with the severity of the disease as ANCA can be found in asymptomatic or healthy individuals (1, 7). The pathogenic T cell epitopes of MPO-AAV have been delineated in experimental models of disease (8).

## Epigenetic and genetic factors of MPO-AAV

The prevalence of AAV has increased in recent years from 48–184 cases to 300–421 cases per million individuals, indicating improved survival of patients (6). Efforts have been put into genome wide association studies (GWAS) to investigate genetic variants and subsets occurring in patients that might be the key towards disease pathogenesis. PR3 is mostly found in Caucasian population whereas Asians such as Chinese and Japanese dominates MPA, indicating the role of different genetic subsets. Studies have supported an association of the disease with non-major histocompatibility complex (MHC) and MHC, though MPO-AAV is mostly associated with MHC II alleles (9). Japanese patients with MPO-AAV are skewed towards DRB1*09:01 being the risk allele (10) whereas Chinese patients are frequently carrying DQA1*03:02, DQB1*03:03 and DRB1*04:05 (11, 12). In particular, MPO-AAV patients carrying DRB1*04:05 exhibit the worst prognosis, with ~50% of patients progressing to end-stage renal failure (ESRF) within 1 year of diagnosis (11).

Other factors including infections, drugs and silica exposure have been associated with disease progression. Patients who are carriers of *S. aureus* are more susceptible to relapse as *S. aureus* infection triggers the disease onset for PR3-AAV. This is because *S. aureus* demonstrates a molecular mimicry to the complementary peptide of PR3_105-201_, and the B cells responds by producing antibodies against this cPR3_105–201_ peptide (13). However, the resulting antibodies were also found to be reactive towards the PR3_105-201_, contributing towards the autoimmunity in patients. Whereas in MPO-AAV, Ooi et al. demonstrated that certain *S. aureus* strain carries a plasmid-encoded 6-phosphogluconate dehydrogenase amino acid sequence (6PGD_391-410_) can trigger anti-MPO immunity due to molecular mimicry (14). Certain drugs such as propylthiouracil (PTU), an antithyroid drug is commonly associated with MPO-AAV even though its exact mechanism is unknown, further contributing towards the risk of certain population to develop AAV (6). Intensity of silica exposure impacts on the development of AAV as well, though its exact mechanism is unknown (15).

Previous studies have identified the immunodominant region of MPO, which tends to be in a ‘hotspot’ region in the heavy chain of MPO. Patients’ sera were used to map these immunogenic epitopes, with identification of shared epitopes between T and B cells further emphasize the importance of the hotspot region through their persistence presence in remission patients. The human chimera to the identified immunogenic MPO epitope in a mouse study by Ooi et. al., MPO_435–453_ lies within the hotspot region (8), whereas clinical studies had identified MPO_393-402_, MPO_437-446_, MPO_447-461_, MPO_479-488_, MPO_717–726_ to be immunogenic (7, 16, 17).

## Diagnosis

Currently, patients are diagnosed based on clinical symptoms; pathology tests (urine and blood) and tissue biopsy (6). However, it must be noted that having a positive ANCA does not necessarily mean the patient has AAV, rather it could be other autoimmune diseases such as lupus nephritis (LN), inflammatory bowel disease (IBD), ulcerative colitis (UC) and autoimmune hepatitis (AIH). Birmingham Vasculitis Activity Score (BVAS) is a well-developed tool that is used by clinicians to quantify the disease activity in patients (18–20). Histologic examination of tissue biopsy remains to be the gold standard in diagnosing AAV. It is crucial to differentiate them with immune complex small vessel vasculitis because it determines the type of treatment and prognosis of patients (6).

## Pathogenesis of MPO-AAV

### Innate immune response

**Figure 1:**
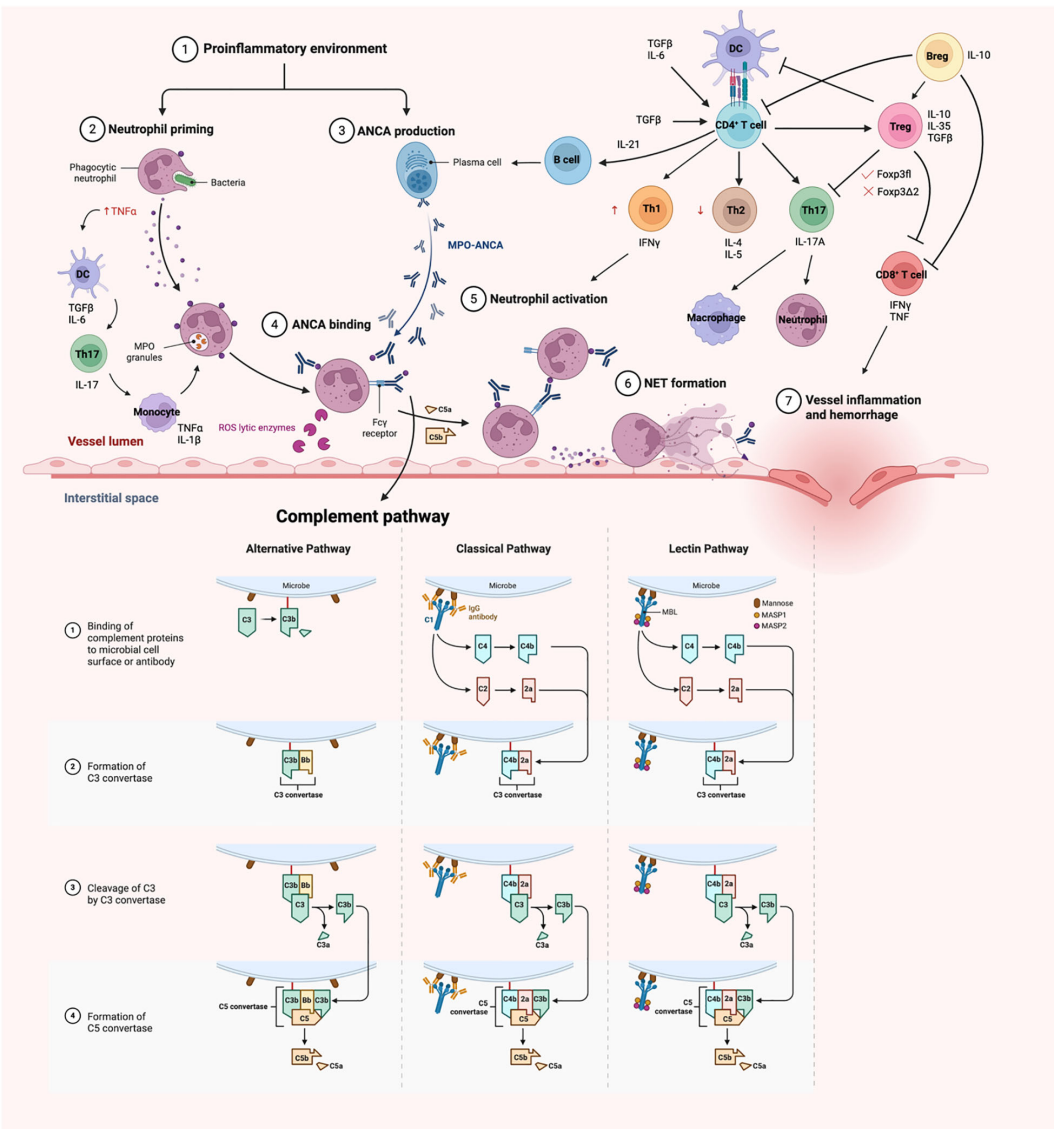
A schematic overview of pathogenesis of MPO-AAV. Neutrophils are primed with TNFα in a proinflammatory environment, resulting in an upregulation of membrane MPO. These neutrophils are targeted by ANCA, hence destroying them in the process, resulting in damage of endothelium lining. The adaptive immune system comes in later and act in a delayed-type hypersensitivity (DTH) fashion, enhancing the disease activity. *Diagrams created with Biorender.com*.

The pathogenesis of AAV is not completely elucidated and the suggested pathways are based on patient and experimental observations (**Figure 1**). The autoantigen, MPO is a sequestered antigen, found in the lysosomes of neutrophils. Hence, a stimulus is required to activate neutrophils to cause an upregulation of membrane bound MPO in neutrophils. Proinflammatory cytokines such as tumour necrosis factor alpha (TNF-α) are usually the trigger for disease onset, which commonly happens in infection. The presence of infectious agent stimulates the release of transforming growth factor beta (TGFβ) and interleukin 6 (IL-6) by dendritic cells (21, 22). ANCAs produced by B cells bind to membrane bound MPO on activated neutrophils. This in turn triggers the naïve T cells to differentiate into T helper 17 (Th17) cells and produce IL-17. IL-17 is a potent proinflammatory cytokine that stimulates the immune system, including the monocytes and macrophages to release TNFα and IL-1β, which further prime the neutrophils during the acute phase of the disease (21, 22). Renal neutrophils are found to be capable of producing proinflammatory cytokines TNFα and IL-17 as well, contributing to the differentiation of Th17 cells. The innate γδ T cells migrate to the site of inflammation and produce IL-17 that contributes to disease development (23).

Studies have shown that TNF-α primed neutrophils upregulates membrane expression of MPO, allowing the interaction between MPO and MPO-ANCA, in which the MPO-ANCA acts as a bridge between the Fcγ receptors and MPO. The FcγR of the adjacent neutrophils can then recognize the Fc portion of these ANCAs, resulting in neutrophil activation, hence releasing reactive oxygen species (ROS) that kills the ‘victim’ neutrophils. This causes the neutrophils to undergo respiratory burst, degranulation, releasing neutrophil extracellular traps (NETosis), apoptosis and necrosis (3).

Furthermore, the FcγR becomes the target of complement, activating the classical and alternate complement pathways, encouraging the destruction of neutrophils. In particular, C5a is the central complement in both pathways and studies had shown an increase in circulating levels of C5a in patients (24). The ablation of the C5a receptor on neutrophils also attenuate the disease phenotype, indicating a probable therapeutic target (25, 26). Despite the importance of FcγR being stated, FcγRIIB plays a role as the only inhibitory Fc receptor that regulates and maintains peripheral tolerance (27). An *in vivo* mouse model showed that the FcγRIIB deficient mice developed more severe glomerular injury. The whole process releases proinflammatory cytokines which attract more neutrophils to the site by adhering to the vessel wall and undergo diapedesis (1–3, 6). Spillage of plasma then happens, and the presence of the coagulation factor encourages the formation of fibrinoid necrosis, ultimately damaging the fragile monolayer endothelium.

### Involvement of the adaptive immune response

#### B cells

B cells are responsible for generating autoantibodies, and some studies suggests that this arises from a deficiency or dysfunction of regulatory T cells (Treg), leading to a loss of immune tolerance. Neutrophils also contribute by releasing B-cell activating factors (BAFF)/B lymphocyte stimulator (BLyS) (28) which stimulate B cell differentiation, proliferation and immunoglobulins (Igs) production. Regulatory B cells (Breg) that express CD5 and is important for IL-10 production, are downregulated in disease. These Bregs play a key role in suppressing activated T cells and supporting Treg differentiation (29, 30). Research has shown that CD5^+^CD24^hi^CD38^+^ B cells are reduced during active disease but return to levels comparable levels to healthy controls (HC) during remission (31). Additionally, CD4^+^ T cells contribute to B cells stimulation through the production of IL-21 (30).

#### T cells

T cells play a critical, non-redundant role in disease pathogenesis (6, 32. 1, 33 *)*. They mediate delayed-type hypersensitivity (DTH) responses, perpetuating inflammation and ultimately leading to tissue destruction (**Figure 1**).

CD4^+^ T helper (Th) cells are central to adaptive immunity, activating B cells, recruiting macrophages and promoting cytotoxic T cell responses, all of which drive autoimmune response. Gan et al. demonstrated that the depletion of MPO-specific CD4^+^ T cells ameliorates GN, highlighting their importance in disease activity (33). Specifically, Th1 cell contributes to the nephritogenic immune responses, where IL-12p40 guides Th1 cells in inducing crescentic GN (34). The balance between Th1 and Th2 cells is critical for maintaining self-immune tolerance (21). Th1 cells drive the severity and progression of autoimmune disease through IFNγ production, while Th2 cells act in opposition (35). Th17 cell is another subset linked to autoimmunity due to its pro-inflammatory nature. Retinoic acid receptor-related orphan nuclear receptor γt (RORγt) is an important transcription factor essential for Th17 cells differentiation, and its absence attenuates GN (36). Both TGFβ and IL-6 encourages the Th17 lineage differentiation, but TGFβ alone supports Treg differentiation (37). Th17 cells produce IL-17 which significantly contributes to disease manifestation, with elevated IL-17 levels exacerbating crescentic GN formation (38). IL-17A not only activates Th17 cells but also enhances neutrophils and macrophages recruitment (39).

In experimental anti-MPO GN mouse models, depletion of MPO-specific CD8^+^ T cells ameliorate kidney injury, as evidenced by reduction in albuminuria, blood urea nitrogen (BUN) and proteinuria (40). Notably, CD8^+^ T cells can mediate glomerular injury in an MPO^+^ environment even in the absence of CD4^+^ T cells, highlighting their independent pathogenic potential (40). These CD8^+^ T cells are major sources of proinflammatory cytokines such as interferon gamma (IFNγ) and TNF. Furthermore, IL7R (CD127) signalling is critical for Teff function, as demonstrated by increased expression in a transcriptome analysis (41).

Besides, toll-like receptors (TLRs) which function to recognize pathogen associated molecular patterns are found to engage in disease pathogenesis. The activation of TLR2 stimulates IL-17A production, promoting Th17 cells activity, while TLR9 enhances anti-MPO driven autoimmunity through Th1 committed lineage pathway (42). In addition, TLR4 is constitutively expressed in glomeruli and its expression is upregulated during GN (43), drive the production of chemokines, CXCL1 and CXCL2 where they serve as the major chemoattractant for neutrophils (43). An experimental murine anti-MPO model further showed that lipopolysaccharide (LPS) can synergize with anti-MPO autoimmunity, exacerbating the NCGN disease phenotype (44). Collectively, these findings suggest that infection and innate immune activation can amplify the anti-MPO driven autoimmunity.

#### Regulatory T cells

A subset of T cells, known as the regulatory T cells (Tregs) are key mediators of self-tolerance and immune homeostasis. The critical role of Tregs was first elucidated in the 1990s by Sakaguchi et. al, where adoptive transfer of CD4^+^CD25^+^ but not CD4^+^CD25^-^ T cells could prevent autoimmune disease in athymic mice (45). The scurfy phenotype observed in mice is linked to mutations in the Forkhead box protein p3 (Foxp3) gene, which is also implicated in the human immunodysregulation polyendocrinopathy enteropathy X-linked (IPEX) syndrome. This syndrome arises from recessive mutations in Foxp3 and results in severe autoimmune manifestations, underscoring the essential role of Foxp3 in Treg function (45).

Foxp3 is a transcription factor uniquely expressed in Treg, but its precise regulatory mechanisms remain incompletely understood. At the genomic level, Foxp3 controls the expression of genes critical for T cell function, including nuclear factor of activated T cells (NFAT) and AML1/RunX1, which are required for effector T cells (Teff) differentiation (46). Foxp3 also interacts with RORγt, inhibiting the differentiation of naïve T cells into Th17, further clarifying its role in MPO-AAV disease mechanism. Notably, only the full length Foxp3 isoform (Foxp3fl) can interact with RORγt, while the exon 2-spliced variant (Foxp3Δ2) cannot (32).

Given their potent immunosuppressive capacity, Tregs are a great therapeutic tool to treat autoimmune diseases. In patients with MPO-AAV, Treg numbers are often elevated, but their suppressive function is frequently imparied (47, 48), potentially due to a reduction in activated Treg (aTreg) characterized by CD45RA^-^Foxp3^high51^. This impairment may also relate to increased expression of the Foxp3Δ2 isoform, which is associated with reduced suppressive function and a higher proportion of resistant CD4^+^ Teff cells within the patient population (47). A recent discovery demonstrated that co-expression of Foxp3fl and Foxp3Δ2 are essential for optimal Treg suppressive capacity (49).

Autoimmune regulator (Aire) is a transcription factor found in lymphoid organs, particularly in medullary thymic epithelial cells (mTECs) of the thymus, where it plays an inevitable role in immune regulation and the establishment of central tolerance. Aire enables mTECs to express a broad array of tissue-specific antigens, facilitating the presentation of self-peptides on major histocompatibility complex (pMHC) molecules to developing T cells. This process ensures that T cells with high affinity TCRs for self-antigens are either deleted or diverted to differentiate into Treg, resulting in a Treg population with a diverse and high affinity T-cell receptor (TCR) repertoire. The importance of Aire is further highlighted through an experimental anti-MPO murine model where Aire deficient mice exhibit more severe disease phenotype due to the escape of autoreactive anti-MPO T cells (50).

Tregs naturally develop in the thymus (nTreg), but they can also be peripherally induced (pTreg) from naïve T cells. While pTreg can be induced transiently in the presence of anti-inflammatory cytokines such as TGFβ and IL-10, they may revert to Teff cells under proinflammatory conditions. TGFβ is crucial for pTreg induction, as it phosphorylates the Smad transcription factors, Smad2 and Smad3, which interact with conserved non-coding sequence (CNS) 1 region in the Foxp3 gene locus, driving Foxp3 expression and thus Treg differentiation. Stable Treg identity is maintained through epigenetic mechanisms. In nTreg, the CpG island in the CNS2 region of the Foxp3 gene which is known as the Treg-specific demethylated region (TSDR) remains hypomethylated, supporting sustained Foxp3 expression and Treg lineage stability even in inflammatory environments. which constitutes towards the promoter (51). The TSDR methylation status distinguishes nTreg from pTreg and is crucial for long-term suppressive function, as TSDR demethylation enables the binding of key transcription factors and preserves cell memory. TSDR comprises of a few functionally crucial genes for Treg differentiation and function, including Foxp3, Ctla4, Il2ra, Ikzf4 and Tnfrs18 (51). On top of the suggested phenotype, CD127 is found to be inversely correlated with Foxp3^+^ cells, and the isolated Treg population is highly purified if included with this marker (52). Therefore, the current best phenotype of Treg is CD4^+^CD25^high^CD127^low^Foxp3^+55^.

Previous pioneer work revealed how Treg functions although its exact working mechanism is still unknown. A primary mode of suppression is the secretion of inhibitory cytokines IL-10, IL-35 and TGFβ, which collectively act to inhibit the activity of effector immune cells (32, 53). IL-35 is a heterodimer formed through the pairing of Epstein-Barr virus-induced gene 3 (Ebi3) and interleukin-12 alpha (Il12a), which is proficient in suppressing T cell proliferation (53, 54). Treg also mediate cytotoxicity through the production of granzymes A and B, which are involved in the direct killing of target cells. The high expression of CD25 on Treg enable them to mop up IL-2, a cytokine essential for T cells survival, thereby depriving Teffs of this growth factor, although IL-2 depletion alone does not fully account for Treg-mediated suppression (53). Another key mechanism involves the generation of pericellular adenosine via the CD73/CD39 ectonucleotidase pathway on Treg (55). This adenosine acts on the adenosine 2A (A2A) receptor on activated T cells, potently inhibiting their activity. Activation of A2A receptor also supresses IL-6 production by Teff while promoting TGFβ generation (56), further shifting the immune response towards regulation rather than inflammation. Furthermore, Tregs interact with dendritic cells (DC) via lymphocyte activation gene (LAG) 3 and cytotoxic T lymphocyte-associated Ag (CTLA)-4 (32, 53). These interactions promote the development of tolerogenic DC, which can produce immunoregulatory enzymes like indoleamine 2, 3-dioxygenase (IDO) that suppresses T cell responses. Odobasic et al. have demonstrated the importance of these tolerogenic DCs in an experimental murine anti-MPO GN model (57).

## Current animal model

**Figure 2:**
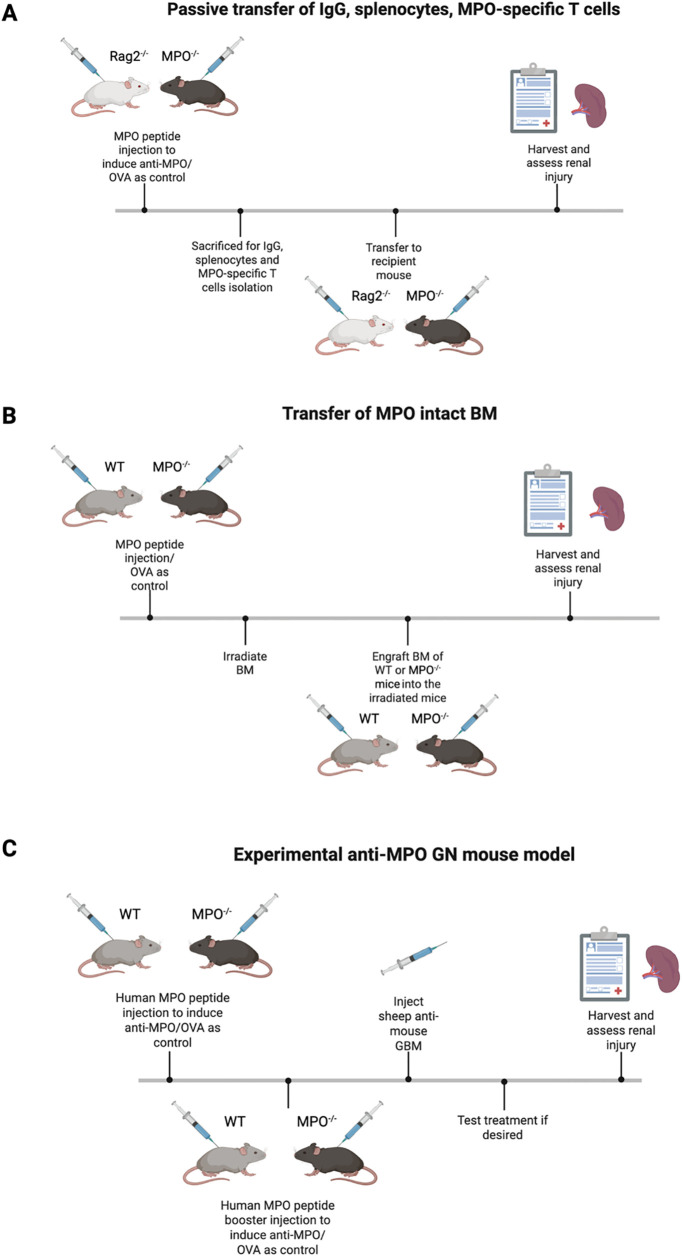
An overview of the current most used mouse models. **(A)** Passive transfer mouse model that showed MPO-ANCA initiates the hallmark of renal injury. **(B)** BM engraftment model further digs into the role of adaptive immune system where MPO-specific T cells enhance renal injury. **(C)** Experimental anti-MPO GN model allows the investigation of MPO-AAV pathogenesis and testing new therapies. *Diagrams created with Biorender.com*.

Animal models are essential for studying diseases like MPO-AAV because they allow researchers to identify therapeutic targets and evaluate potential treatments *in vivo*. However, most current MPO-AAV models primarily replicate the acute phase of disease rather than full remission, highlighting the need for improved models that better capture the complexity of human disease. To summarize, there are currently 4 ways to induce MPO-AAV in mice (refer to **Figure 2**).

### Passive transfer of IgG, splenocytes, MPO-specific T cells into mice

The foundational anti-MPO GN mouse model was developed by Xiao et al. and has been instrumental in understanding MPO-AAV pathogenesis (58, 59). In this model, MPO knockout (MPO^-/-^) and recombinase-activating gene-2-deficient (Rag2^-/-^) mice, where the Rag2^-/-^ mice lack both T and B cells are first immunized with MPO to induce anti-MPO immune responses. IgG is then isolated from these immunized mice and injected into wildtype (WT) or Rag2^-/-^ mice. This transfer reliably induces focal necrotizing GN, providing direct evidence that anti-MPO IgG alone can cause renal injury. Disease severity increases when splenocytes of the MPO-immunized MPO^-/-^ mice are transferred into WT and Rag2^-/-^ mice (58). These recipient mice develop severe NCGN, with marked elevation of BUN and serum creatinine, indicating significant kidney dysfunction. The disease can be further exacerbated by introducing proinflammatory stimuli such as LPS, resulting in even more severe pathology. Despite the utility of this model in establishing the pathogenicity of anti-MPO antibodies and immune cell, they have limitations. Notably, glomerular immune complex deposition is commonly observed, which differs from the pauci-immune pattern in human cases of MPO-AAV. Therefore, while these models are invaluable for dissecting disease mechanisms and testing therapies, further refinement is necessary to more accurately reflect human disease.

### Transfer of MPO intact bone marrow

Another strategy involves first immunizing MPO^-/-^ and WT mice with MPO, followed by irradiation (59, 60). Bone marrow from naïve WT or MPO^-/-^ mice is then transplanted into these immunized recipients to repopulate their immune cells. Only mice receiving WT bone marrow but not those receiving MPO^-/-^ bone marrow develop NCGN after transfer. This finding underscores the necessity of MPO+ neutrophils in peripheral blood of recipient mice for disease development post engraftment. These results suggest that immune cells, particularly neutrophils are the pathogenic targets for anti-MPO IgG (60).

### Active immunization of MPO inducing experimental GN

The experimental anti-MPO GN murine model is particularly valuable as it enables the study of loss of tolerance and the development of active autoimmunity. This model also facilitates the identification of potential therapeutic targets by allowing reestablishment of immune tolerance, which is not possible in the passive transfer model. In this approach, WT and MPO^-/-^ mice are sensitized with human MPO to induce anti-MPO antibodies (59, 61). These mice are then injected with sheep anti-mouse glomerular basement membrane (GBM) ten days later to activate neutrophils and deposit MPO in the glomeruli, thereby inducing experimental MPO-ANCA. The use of a minimal dose of sheep anti-mouse GBM is important as it triggers neutrophils influx into the glomeruli without causing significant anti-GBM disease, which would confound the results. Importantly, only MPO-sensitized mice develop diseases, confirming the requirement for both anti-MPO immunity and MPO deposition in disease pathogenesis. Ovalbumin (OVA) was also included as control as an irrelevant antigen confirm the specificity of the model (61). This model advances our knowledge in characterizing the role of CD4^+^ and CD8^+^ T cells as well as B cells in disease pathogenesis.

Although the above-mentioned animal models are well-established for the study of MPO-AAV, exploring options beyond them is necessary to facilitate with the understanding of later phase of disease. Humanized mouse model and organoids are promising next steps for the advancement of preclinical models (62, 63). Harnessing immunodeficient mice to allow engraftment of human cells creates a 3D environment for the study of cells and tissues interactions, however, it is usually expensive and is met with the common limitations of cross-species difference. On the other hand, the cost-effective organoids-based approaches is attractive as it aligns with the reduction usage of animals but retaining the capability for inter-species translation. Kidney is a complex organ and the development of 3D organoids is still on-going with efforts being put into mimicking the kidney environment with reproducible conditions (64). Nevertheless, more research is still required for the development of appropriate preclinical models to assist with the study of chronic disease pathogenesis and treatment targets.

## Treatment

### Current therapy

Treatment for AAV is segregated into two phases, that are induction of remission and maintenance therapy. The current standard treatment for severe AAV is the combination of glucocorticoids; either prednisone with cyclophosphamide (CYC) or with rituximab (RTX) (65, 66). Oral CYC is associated with greater toxicity due to prolonged drug exposure, thus pulse intravenous (i.v.) CYC is preferred, despite a higher relapse risk. This preference is supported by evidence showing that patients receiving i.v. CYC experience less renal impairment and fewer cases of leucopaenia (67, 68). CYC toxicity arises from its broad immunosuppressive effects, often lead to opportunistic infections such as *Pneumocystis jiroveci* pneumonia, haemorrhagic cystitis, malignancy, and gonadal failure resulting in infertility (68). Meanwhile, RTX is an anti-CD20 monoclonal antibody drug that depletes B cells, thereby preventing ANCA production. RTX is preferred if CYC overdose is a concern, or in patients prone to relapse. Additionally, RTX had been shown to enhance Treg immunomodulatory capacity by inducing B cell apoptosis, though B cell depletion can result in hypogammaglobulinaemia that leads to immune suppression (69).

To minimize cumulative CYC exposure, alternate therapies such as methotrexate (MTX) are considered in early disease, although longer treatment is required and relapse rates are higher compared to CYC (70, 71). Maintenance therapy may involve MTX or azathioprine (AZA), both of which are noninferior to CYC and associated with lesser adverse events (66, 72). Leflunomide and mycophenolate mofetil (MMF) are less commonly used as leflunomide is associated with higher frequency of adverse events (73, 74), whereas in the case of MMF, showed lower efficacy than AZA except in MPO-AAV patients experience less severe renal disease (75, 76).

Given the toxicity of long-term immunosuppressive therapy, biologic agents are being explored. TNFα blocker like etanercept is tested in GPA patients though not proven to be effective in MPO-AAV patients (66, 77). On the contrary, plasma exchange is considered adjunctive for severe cases with pulmonary and renal involvement (78, 79), though recent study shows no superiority over standard treatment (80). Long-term outcome on these patients, however, have shown promising results as they require lower steroid dosage for maintenance therapy, reducing toxicity. Avacopan (CCX168), a C5a inhibitor, has shown promise in replacing glucocorticoids for maintenance therapy (81, 82), potentially reducing morbidity and mortality in AAV patients (83). Patients are spared from higher dose of steroids and safer for renal recovery. Low dose IL-2 is another emerging option, selectively stimulating Treg cells to modulate immune response (84). A recent study employing spatial transcriptomics and digital pharmacology had identified ustekinumab, a human monoclonal antibody which target IL-12 and IL-23 to be effective in disease remission (85). It potently inhibits the dominated Th1/Tc1 and Th17/Tc17 cells in inflamed kidneys as seen in patients, suggesting the use of personalized therapy through combination of current available drug with rapid immune profiling.

### Future therapy

While current standard treatment is well-defined with mature clinical trials results supporting in place (**Table 1**), retaining kidney function is the top priority and with several events of relapse, patients eventually develop end-stage renal failure. Therefore, exploring alternative therapeutic options is important, and recent success in emerging cell-based therapies that offer personalized treatment with fewer side effects through direct target of disease mechanism.

**Table 1:** Summary of current available therapy associated with their clinical trials studies.

Biologics	Disease phase	Clinical trial no.	Clinical trial phase
Oral vs IV CYC	Induction of remission	NCT01697267	RITUXVAS
Pulse vs continuous CYC	Induction of remission	NCT00430105	CYCLOPS
MMF vs CYC	Induction of remission	NCT00301652	MYCYC
MMF	Induction of remission for relapse	NCT00103792	Phase 3
Rituximab	Induction of remission	NCT00104299	RAVE Phase 2 Phase 3
Plasma exchange	Induction of remission	NCT00987389	PEXIVAS Phase 3
CCX168	Induction of remission	NCT02994927	ADVOCATE Phase 3
Belimumab	Maintenance	NCT01663623	BREVAS Phase 3
Rituximab	Maintenance	NCT02433522	MAINRITSAN Phase 1-3

#### Adoptive cell transfer

Previous attempts on harnessing the potent immunosuppressive capacity of Treg have led to various studies evaluating their efficacy in treating autoimmune diseases and preventing graft rejection. *Ex vivo* expanded polyclonal Treg have been tested in multiple clinical settings and have shown promise in inducing immune tolerance. This process involves isolating Tregs from patients’ peripheral blood mononuclear cells (PBMCs), expanding them *in vitro*, and then reinfusing them into the patients.

Multiple studies have demonstrated that adoptive transfer of Tregs attenuates symptoms of immune-mediated conditions, such as asthma (86), graft versus host disease (GvHD) (87) and several autoimmune diseases including type 1 diabetes (T1D) (88), multiple sclerosis (MS) (89) and autoimmune hepatitis (AIH) (90). For example, a phase 1 clinical trial evaluated the polyclonal Treg therapy in paediatric T1D patients, with results indicating therapeutic effectiveness following the infusion of two doses of polyclonal Tregs (91). However, similar benefit was not observed in adult trials, where no significant efficacy was detected (88). This discrepancy may be due to differences in immune system maturity between children and adults, leading to varied responses to therapy.

Another possible explanation is that polyclonal Treg may function differently in inducing peripheral immune tolerance. One study suggested that polyclonal Treg might inhibit the migration of Teff cells to specific tissue sites, whereas antigen-specific iTreg can act directly at the target site, potentially providing more precise immune regulation (92). This distinction highlights the potential advantages of using antigen-specific Tregs, especially since autoimmune diseases are often diagnosed only after tissue damage has occurred. In such cases, Teff cells may already have infiltrated the target site, making site-specific intervention by engineered Tregs a more effective therapeutic approach.

#### CAR-T cell therapy

Chimeric antigen receptor (CAR)-T cell therapy is an FDA-approved treatment that specifically targets CD19, a B cell antigen, and has demonstrated significant efficacy in the management of B cell leukaemias and lymphomas (93, 94). However, the application of CAR-T cell therapy to solid tumours has met with limited success, primarily due to the unique challenges posed by the tumour microenvironment and tumour biology (95, 96).

**Figure 3:**
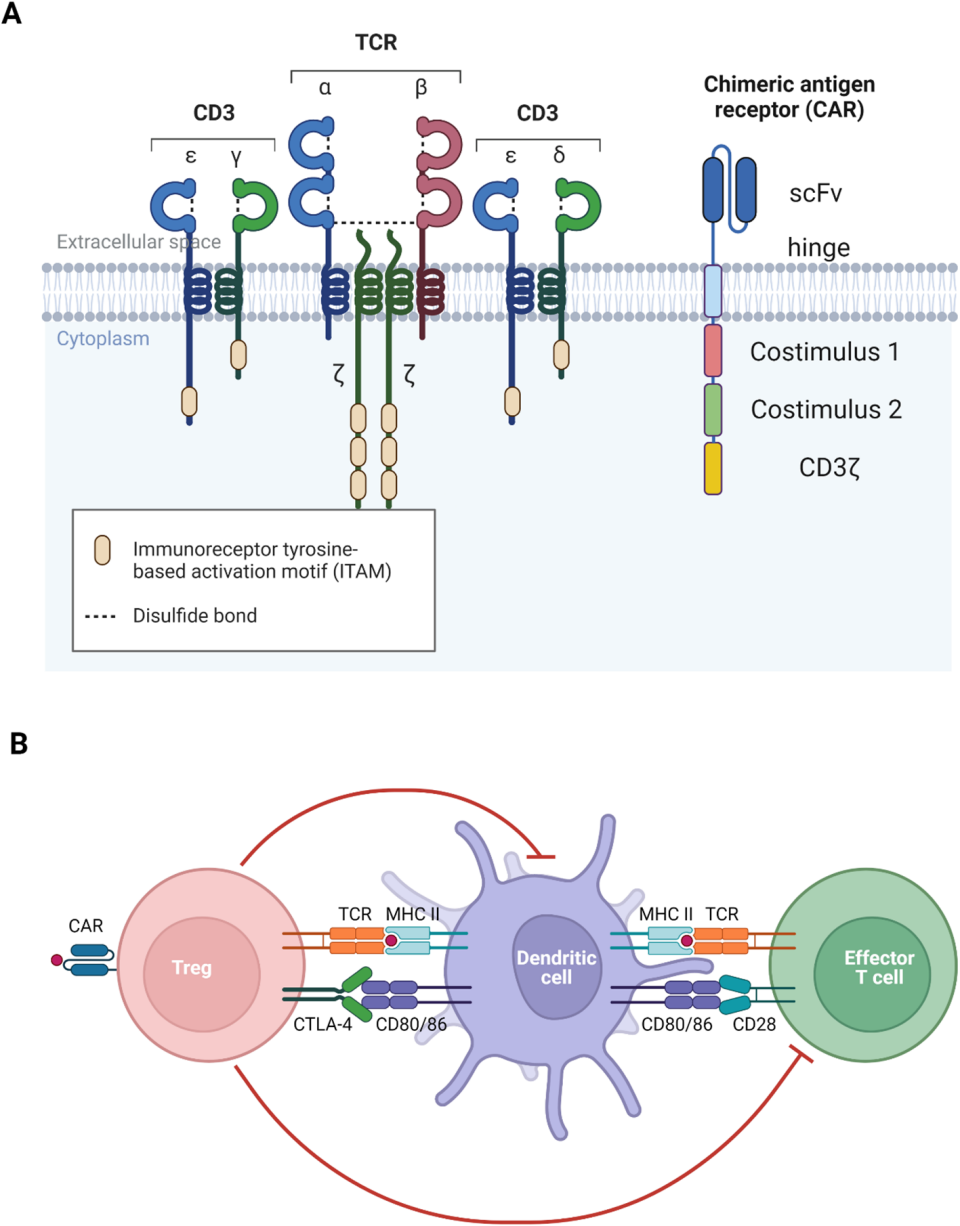
Visualization of TCR and CAR. **(A)** The main difference between TCR and CAR is that TCR recognizes MHC-bound peptide but CAR recognizes free antigen. **(B)** Both TCR and CAR function to activate the generated antigen-specific Treg to recognize self-antigen to induce immune tolerance. *Diagrams created with Biorender.com*.

The engineering of CAR-T cells involves the introduction of a synthetic receptor, the chimeric antigen receptor, which is composed of several key domains. The extracellular portion consists of a single-chain variable fragment (scFv), derived from the variable region of antibody heavy and light chains (refer to **Figure 3**), which confers antigen specificity (97, 98). A flexible spacer region links the scFv to a transmembrane domain, allowing for optimal antigen binding. The transmembrane domain anchors the receptor within the T cell membrane and connects to intracellular signalling domains. The first-generation CARs utilized only the CD3ζ signalling domain, resulting in weak T cell activation and limited therapeutic persistence. Subsequent generations incorporated additional costimulatory molecules, such as CD28 or 4-1BB (CD137), which markedly enhance CAR-T cell activation, persistence, and overall therapeutic efficacy.

Despite these advances, several barriers hinder the effectiveness of CAR-T cell therapy in solid tumours. A major challenge is the limited infiltration of CAR-T cells into the tumour site, which is influenced by the tumour’s structural complexity and the surrounding extracellular matrix. Additionally, the tumour microenvironment is often immunosuppressive, characterized by low oxygen levels, high acidity, and nutrient scarcity, all of which impair CAR-T cell function and survival. Tumour heterogeneity, whereby cancer cells exhibit diverse antigen expression profiles, further complicates the targeting of all malignant cells by CAR-T therapy.

The success of CAR-T cell therapy in haematologic malignancies has spurred interest in extending its application to other disease areas (98). For example, preclinical and early clinical studies are exploring the use of CAR-T cells for the treatment of autoimmune diseases, such as systemic lupus erythematosus (SLE) (NCT03030976) and carcinoembryonic antigen (CEA)-specific CAR-Treg to treat ulcerative colitis (UC) (99). Additionally, CAR-T cell approaches are being investigated to induce immune tolerance in patients with haemophilia A, with the aim of enabling sustained recombinant Factor VIII therapy without the risk of inhibitor formation. Other autoimmune diseases including myelin-oligodendrocyte glycoprotein (MOG)-specific CAR-Treg to treat MS (100) and Dsg-3 chimeric autoantibody receptor (CAAR)-T to treat pemphigus vulgaris (PV) (101). This review will only cover the aspects of autoimmune diseases for the sake of comparison between treatments in MPO-AAV. CAR-Treg is easier to produce and convenient to use as it is independent of MHC antigen recognition, hence it does not select patient population for a specific HLA to use (**Table 2**).

**Table 2:** Comparison between the cell-based therapies.

Cell-based therapy	Advantages	Disadvantages
CAR-Treg	• Not HLA specific, can be used for any population with the specified disease • Bispecific targets of CAR-T cells was developed and proved to be potent	• Requires higher antigenic dose for T cell activation • CAR molecule is not endogenous to human, might trigger immunity against CAR, destroying the cells. • Cells are less stable; longevity of cells is a problem
TCR-Treg	• Requires only small antigenic dose to activate the T cells • TCR is endogenous to human, unlikely to trigger immunity against the cells • TCR-T cells are more stable	• HLA and antigen specific

#### TCR-Treg therapy

Since the manifestation of MPO-AAV also involves the autoantigen recognition of immune cells presenting processed MPO on MHC II (refer to **Figure 3**), TCR-Treg is suited to use to target these autoreactive cells. The approach involves directing Treg to recognize MHC II-bound MPO, which can be achieved through gene transfer of T cell receptor (TCR) specific to the epitope of interest.

Although further studies are required to confirm the efficacy of TCR-Treg, several *in vitro* studies provide foundational insights. T1D, a highly prevalent autoimmune disease, serves as a notable reference point for such research. An *in vitro* study on T1D had employed TCR-Treg specific for islet antigen 2 (IA2) and insulin, with influenza-specific TCR-Tregs included as controls to assess specificty (102). The suppressive potency of these TCR-Tregs was evaluated by co-culturing them with either wild-type or antigen-presenting cells loaded with their respective peptides. Results demonstrated that the generated TCR-Tregs are highly immunosuppressive in the presence, but not absence of their cognate peptides (102). Conversely, patients with Haemophilia A require constant intake of recombinant FVIII (rFVIII) as they lack the critical coagulation factor FVIII, however, rFVIII is not native to patients’ immune systems, thus causing gradual destruction of rFVIII (103, 104). To address this, studies have introduced FVIII-specific TCR-Treg into patients, showing successful suppression of immune responses against FVIII-specific Teff cells (103). Additionally, another study had explored the use of myelin-basic protein (MBP)-specific TCR-Treg to investigate the efficacy in treating MS (105). Despite the need for further validation, these findings collectively highlight the therapeutic potential of antigen-specific TCR-Treg in a range of autoimmune conditions.

Previous studies have successfully determined the immunodominant epitope of MPO that triggers autoimmune activation, with disease severity and prevalence are HLA-linked. The importance of HLA-DRB1*04:05 and HLA-DQA1*03:02, DQB1*03:03 in Chinese patients whereas HLA-DRB1*09:01 in Japanese patients were well elucidated. This attracts molecular studies to be performed on these disease-linked HLAs, which could possibly open the doors to enhance the development of better cell-based therapy. HLA-DRB1*04:05 is the most severe related HLA class II molecule found in patients that usually progress into ESRF within six months even with existing treatment. Thus, initiating the alternative cell-based therapy with HLA-DRB1*04:05 would be a major step towards the development of next-generation personalized therapy (**Table 2**).

## Challenges in TCR-Treg production

Cell-based therapy represents a promising frontier in individualized immunotherapy, yet the high production cost remains a significant barrier to widespread clinical adoption. The process is resource-intensive, requiring the collection of blood, isolation of Treg, transduction and expansion of these cells, and ultimately their infusion into patients. To address scalability and cost, one proposed solution is the use of platforms such as Lonza, which can be adopted by laboratories that mimic clean room environments, thereby commercializing production while maintaining the Good Manufacturing Practice (GMP) standards. A major safety concern in TCR-Treg therapy is the potential for the introduced transgenic TCR to mispair with the endogenous TCR, leading to the formation of hybrid receptors that may trigger harmful autoimmune responses. To mitigate this risk, clustered regularly interspaced short palindromic repeats (CRISPR)-based knockout of the endogenous TCR prior to gene transfer has been explored. Studies have shown that this approach enhances the expression of the transduced TCR, resulting in improved function of engineered T cells (106). By also knocking out endogenous HLA presenting on the Tregs, the product could be possibly made off-shelf, significantly improving the feasibility of the therapy. Although autologous T cell therapy is more favourable due to unlikely event of graft-versus-host disease (GvHD) in patients, it is less available to rural area and is often required to be operated at a more centred venue for product production, storage and delivery. This makes off-shelf product to be more convenient as it does not require patients’ cells and with proper management, they can be delivered to more rural areas for infusion. Knocking in the TCR of interest using the same method can therefore create super-Tregs. These super-Tregs can be further produced through induction from peripheral T cells to iTreg; and through CRISPR-based demethylation of the TSDR region of T cells (107, 108), can create a more stable Treg phenotype, overcoming the limit on the Tregs number since they occur in small amount naturally. The pipeline for Treg cell therapy production includes isolation, expansion, storage, transport, infusion into the patients. The toughest part is the scalability and cost as the naturally existing Tregs occur in small numbers in human body. But with the above-mentioned gene editing method, CD4^+^ T cells can be isolated in larger numbers and expanded. The study of Tregs stability is crucial as Tregs can still convert to Teffs under pro-inflammatory environment, hence, *in vivo* humanized model is required for not only to study the functionality of the engineered Tregs, but also to determine their longevity and stability.

There are several current Treg manufacturing protocols, and no method is proven to be superior to one another. A standardized protocol is yet to be developed as little is known about how the different manufacturing options can cause different patients’ outcome, though they are not supposed to differ significantly to each other. Briefly, the enriched Treg population can be expanded in presence of rapamycin to maintain Treg phenotype and prevent Teff expansion. Other methods including bead-based enrichment and flow cytometry-based selection to expand then isolate is considered as well. A recent GMP certified protocol approved by the Spanish authority can be considered as well (109), and using this as a foundation to further modify the product can help with the establishment of Treg product. As the study only used natural Treg as the source for amplification and subsequent product development, the iTreg product is yet to be investigated.

There are several challenges regarding cell-based therapies, especially the optimal treatment regimen for patients. Given the nature of complexity of autoimmune diseases, current landscape involves infusing the engineered Tregs into patients for induction of remission. However, the longevity of these infused cells remains unknown, as a recent CAR-T study in an autoimmune disease, idiopathic inflammatory myositis indicate patients may relapse within a year (110). It is still unknown whether the cell-based therapies can be given for maintenance therapy. Options for therapy include one-time induction until disease recurrence or utilizing a higher initial dose followed by lower, regular doses for maintenance. Patient outcomes are still measured using standards like the BVAS score, which provides clear criteria for diagnosis and disease activity. A key advance would be the elimination of steroids as part of therapy, greatly improving quality of life and disease manageability. Autoimmune diseases are challenging and long-term, therefore, robust and comprehensive studies including long-term clinical trials spanning 5–10 years are necessary to fully understand patient outcomes and refine future treatment strategies.

## Future directions

MPO-AAV with HLA-DRB1*04:05 garners particular interest because, despite its rarity, it is associated with the poorest 5-year survival among MPO-AAV. Cell-based therapies using engineered Treg are attractive for MPO-AAV as they may circumvent the broad immunosuppression and associated side effects observed with conventional treatments such as CYC or RTX combined with glucocorticoids. Two main strategies are under investigation, which are CAR-Treg and TCR-Treg therapies. Of these, TCR-Treg which recognize antigen-MHC complexes, may offer superior efficacy in MPO-AAV due to their MHC-dependence in ensuring the specificity of the developed therapy to minimize possible side effects. Although clinical trials using CAR-Treg and TCR-Treg therapies in MPO-AAV have not yet commenced, both *in vitro* and *in vivo* studies in other autoimmune diseases support their feasibility and therapeutic potential. This is further underscored by promising results in related models, such as MOG-specific CAR-Treg and MBP-specific TCR-Treg for MS as well as the FVIII-specific CAR-Treg and FVIII-specific TCR-Treg to tolerate rFVIII in Haemophilia A patients. In summary, while TCR-Treg therapy holds great promise for the treatment in refractory autoimmune diseases, its clinical translation will require overcoming challenges related to manufacturing scalability, cost, and safety. Advances in gene editing technologies and optimized manufacturing protocols are critical next steps for bridging these therapies into routine clinical practice.

This also allows exploration of other similar diseases of vasculitis in the broader terms for the application of the Treg-based therapy. Since other vasculitis involving big and medium vessels like giant cell arthritis involves a more systemic disease rather than organ-specific, a potent alternate therapy to the corticosteroid’s regimen is required for the patients’ quality of lives and prognosis (111). The involvement of adaptive immune system points to the vital role of Tregs to be developed as a possible next-generation personalized therapy.

## Literature searches and article selection

Clinicaltrials.gov (Pubmed) was used for clinical trial searches on current treatment in ANCA-AAV using the keyword ANCA associated vasculitis. The search was further filtered to include only interventional, completed and active, but not recruiting participant studies. 73 studies were filtered out and only studies covering MPA were selected. The Google search engine was used to select top 30 articles with keywords including pathogenesis of MPO-AAV, Treg function, therapy for MPO-AAV in separate occasions. Related references within the selected articles were further studied and included as part of review writing.
